# Supplementary material for: An empirical study of software ecosystem related tweets by npm maintainers
Source: PeerJ Comput Sci. 2024 Jan 17;10:e1669. doi: 10.7717/peerj-cs.1669 (PMC10803008; doi:10.7717/peerj-cs.1669)
Supplement: Supplemental Information 1 [file peerj-cs-10-1669-s001.zip › Replication Package/Figures/methodology.pptx]

## Slide 1
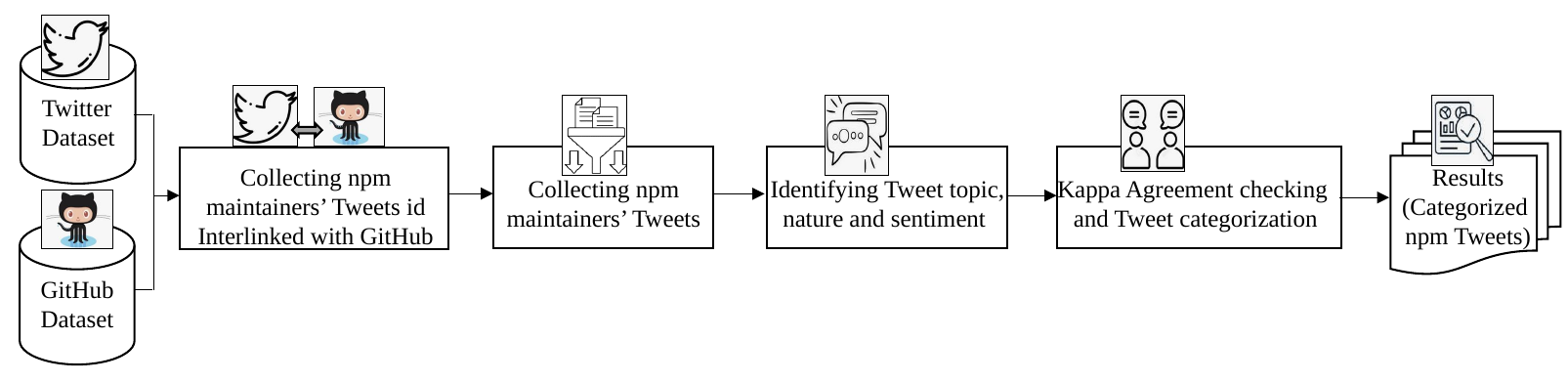

Twitter
Dataset
Results
(Categorized
npm Tweets)
Collecting npm maintainers’ Tweets
 Identifying Tweet topic, nature and sentiment
Kappa Agreement checking
and Tweet categorization
Collecting npm maintainers’ Tweets id
Interlinked with GitHub
GitHub
Dataset
