# Supplementary figures and images for: An empirical study of software ecosystem related tweets by npm maintainers
Source: PeerJ Comput Sci. 2024 Jan 17;10:e1669. doi: 10.7717/peerj-cs.1669 (PMC10803008; doi:10.7717/peerj-cs.1669)

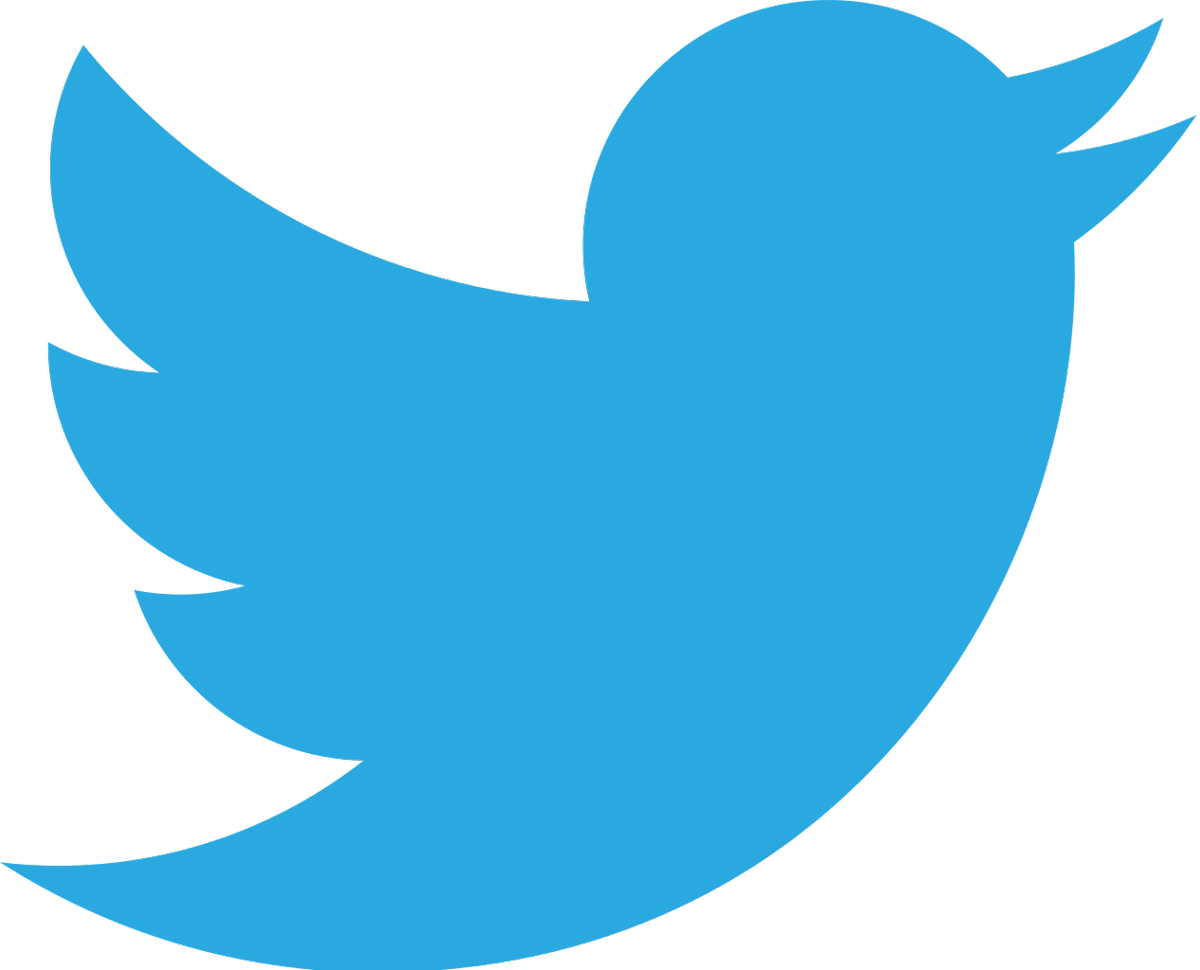

Supplement: Supplemental Information 1 [file peerj-cs-10-1669-s001.zip › Replication Package/Figures/mask.png]
